# Probiotic Properties of *Alcaligenes faecalis* Isolated from *Argyrosomus regius* in Experimental Peritonitis (Rat Model)

**DOI:** 10.1007/s12602-021-09767-7

**Published:** 2021-03-13

**Authors:** A. I. Gutiérrez-Falcón, A. M. Ramos-Nuez, A. Espinosa de los Monteros y Zayas, D. F. Padilla Castillo, M. Isabel García-Laorden, F. J. Chamizo-López, F. Real Valcárcel, F. Artilles Campelo, A. Bordes Benítez, P. Nogueira Salgueiro, C. Domínguez Cabrera, J. C. Rivero-Vera, J. M. González-Martín, J. Martín Caballero, R. Frías-Beneyto, Jesús Villar, J. L. Martín-Barrasa

**Affiliations:** 1 Group of Fish Health and Infectious Diseases, Universitary Institute of Animal Health and Food Safety (IUSA), University of Las Palmas de Gran Canaria, Carretera de Trasmontaña s/n, 35416 Arucas, Spain; 2 CIBER de Enfermedades Respiratorias, Instituto de Salud Carlos III, Monforte de Lemos 3-5, Pabellón 11, 28029 Madrid, Spain; 3 Multidisciplinary Organ Dysfunction Evaluation Research Network, Research Unit, Hospital Universitario de Gran Canaria Dr. Negrín, Barranco de la Ballena s/n, 35019 Las Palmas de Gran Canaria, Spain; 4 Morphology Department, Universitary Institute of Animal Health and Food Safety (IUSA), Universidad de Las Palmas de Gran Canaria. Arucas, Las Palmas, Spain; 5 Microbiology Department. Hospital,, Universitario de Gran Canaria Dr Negrín, Barranco de La Ballena S/N, 35019 Las Palmas de Gran Canaria, Spain; 6 Clinical Biochemistry Department, Hosital Universitario de Gran Canaria Dr Negrín, Barranco de La Ballena S/N, 35019 Las Palmas de Gran Canaria, Spain; 7 Pathology Service. Hospital, Universitario de Gran Canaria Dr Negrín, Barranco de La Ballena S/N, 35019 Las Palmas de Gran Canaria, Spain; 8 Statistics Service. Research Unit, Hospital Universitario de Gran Canaria Dr Negrín, Barranco de La Ballena S/N, 35019 Las Palmas de Gran Canaria, Spain; 9 Barcelona Biomedical Research Park (PRBB), Barcelona, Spain; 10 Comparative Medicine, Karolinska Institutet, Stockholm, Sweden; 11 Animal Facility, Research Unit, Hospital Universitario de Gran Canaria Dr Negrín, Barranco de La Ballena S/N, 35019 Las Palmas de Gran Canaria, Spain

**Keywords:** *Alcaligenes faecalis*, Peritonitis, Probiotic, *Argyrosomus regius*, Rat, *Escherichia coli*

## Abstract

A strain of *Alcaligenes faecalis* A12C (*A. faecalis* A12C) isolated from *Argyrosomus regius* is a probiotic in fish. Previous experiments showed that *A. faecalis* A12C had inhibitory effects on the growth of multidrug-resistant bacteria. We aimed to confirm whether *A. faecalis* A12C is safe and has adequate intestinal colonization in experimental rats, and evaluate its efficacy in an animal model of peritonitis*.* We used 30 male rats, randomly divided into 6 groups (*n* = 5): three groups (HA7, HA15, HA30) received *A. faecalis* A12C in drinking water (6 × 10^8^ CFU/mL) for 7 days, and three control groups received drinking water only. All groups were evaluated at 7, 15, and 30 days. Survival after *A. faecalis* A12C administration was 100% in all groups. Mild eosinophilia (1.5%, *p* < 0.01) and increased aspartate aminotransferase (86 IU/L, *p* < 0.05) were observed in HA7, followed by progressive normalization. No histological signs of organ injury were found. We observed significant *E. coli* decline in faeces, parallel to an increase in *A. faecalis* A12C at 7 days. *E. coli* had a tendency to recover initial values, while *A. faecalis* A12C disappeared from the intestinal microbiota at 30 days. To evaluate its efficacy against peritonitis, we studied two additional groups of animals: IA group pretreated with *A. faecalis* A12C before *E. coli* intra-abdominal inoculation, and IC group inoculated with no *A. faecalis* A12C. We found an increase in C-reactive protein, alanine aminotransferase, urea, and eosinophils in IC animals when compared with IA. Peritonitis was more evident in IC than in IA animals. Our findings suggest that *A. faecalis* A12C altered clinically relevant parameters in sepsis and was associated with a lesser spread of infection.

**Supplementary Information:**

The online version contains supplementary material available at 10.1007/s12602-021-09767-7.

## Introduction

The abdomen is the most common source of sepsis and is associated with an unacceptably high morbidity and mortality [1]. Sepsis is defined as a life-threatening organ dysfunction caused by a deregulated host response to infection [2]. Despite developments in antisepsis and antibiotic prophylaxis, septic complications are common in surgical patients. Many postoperative infections are caused by intestinal bacteria, and the intestinal barrier and patient's indigenous intestinal microbiota facilitate the development of complications such as peritonitis [3, 4] that could be fatal [5]. *Escherichia coli* (*E. coli*) is the most common pathogen in abdominal sepsis, in both human and rodents [6–9]. Direct administration of *E. coli* is one of the classic experimental models of peritonitis in rats [4].

Probiotics are living microorganisms that confer health benefits to the host through an interaction with the intestinal microbiota and the immune function when administered in adequate doses [10]. A variety of species of probiotics (including *Bifidobacterium spp*, *Lactobacillus spp*, *Enterococcus spp*, *Streptococcus spp*, *Bacillus spp*, and yeasts) have shown to benefit human and animal health both in vivo and in vitro [9, 11–13]. There are increasing numbers of studies evaluating the benefits of new candidate bacterial species as probiotics [14–18]. Studies with probiotics strains of *Lactococcus spp*, *Enterococcus spp*, *Streptococcus spp*, *Vibrio spp*, *Bacillus spp*, *Pseudomonas spp*, and *Aeromonas spp* [19–21] have revealed benefits for their natural hosts, by increasing immune response against an specific pathogen, production of inhibiting compounds, or competing for fixation sites.

The use of probiotics and immune stimulants has had a remarkable increase in aquaculture due to the trend of reducing the use of antibiotics. Currently, probiotics and immune stimulants are considered candidates to control fish diseases. In pilot studies from our group, strains of *Alcaligenes faecalis*, isolated from *Argyrosomus regius* gills, have shown in vitro excellent conditions as potential probiotic in fish due to inhibitory activity against the main bacterial fish pathogens [22]. These in vitro results have been confirmed in several in vivo experiments, reporting a potent protective effect after infection with the marine pathogen *Vibrio anguillarum*. In preliminary experiments with several multidrug-resistant bacteria from hospital origin, we found that *Alcaligenes faecalis* A12C (*A. faecalis* A12C) had inhibitory effects on the growth of *E. coli*, *Klebsiella pneumoniae*, and *Enterobacter cloacae*. Antibiotics are the first option to control infectious diseases due to their rapid action and availability. However, despite of being an effective strategy in the beginning, the negative effects on environmental and public health justify the need of developing new strategies for the prevention and treatment of bacterial infections in both human and veterinary medicine. Based on these pilot studies, we aimed to evaluate the potential therapeutic effect of *A. faecalis* A12C in an experimental, clinically relevant animal model of peritonitis.

## Materials and Methods

### Animals

This study was approved by the Local Animal Ethics Committee, Hospital Universitario Dr. Negrín, Las Palmas de Gran Canaria, Spain, and followed the recommendations of the European Commission (2010/63/EU), and the Spanish Legislation (Law 53/2013) for the protection of animals for scientific purposes. We used forty-two male Crl:Sprague–Dawley® (SD) rats of 12 weeks of age, obtained from a breeding colony kept under semi-barrier conditions from the animal facilities of Hospital Universitario de Gran Canaria, Dr. Negrín, were a third generation of a colony from Charles River (Barcelona, Spain). Rats were housed in pairs in mini-aisled cages of 1500 cm^2^ (480 × 375 × 210 mm) (Tecniplast, Buguggiate, Italy) with enrichment consisting of an igloo and some nesting material. Cage change was undertaken twice a week. Rats were fed with a diet consisted of rat chow pellets (Teklad® Global 14% Protein Rodent Maintenance Diet, Harlan, Barcelona, Spain) and drinking water (Fonteide®, S/C de Tenerife, Spain) were available ad libitum. The light/dark cycle was 12/12 h. Room temperature was maintained at 21 ± 1 °C, and relative humidity was 55 ± 5% with an air exchange rate of 15 times/h. All the rats were acclimatized for 21 days and were determined to be healthy on the basis of individual physical examinations, and pathogen-free based on the results of routine microbiological screening performed on the colony in accordance with European recommendations [23].

### Preparation of *A. faecalis* A12C for Administration to Rats

The probiotic strain *A. faecalis* A12C was isolated from *Argyrosomus regius* gills at the Instituto de Sanidad Animal y Seguridad Alimentaria of the University of Las Palmas de Gran Canaria. The strain was identified at the Microbiology Department of the Hospital Universitario de Gran Canaria Dr Negrín by means of matrix-assisted laser desorption ionization time-of-flight mass spectrometry (MALDI-TOF MS) system (Vitek®MS, Biomerieux, Madrid, Spain). *A. faecalis* A12C strain cultures were stored at − 80 °C with 20% glycerol (v/v) addition in Brain Heart Infusion broth (PanReac-AppliChem, Darmstadt, Germany). Fresh cultures were made prior the assays, and the strain was aerobically incubated in Trypticase Soy with 5% sheep blood (Becton Dickinson, Franklin Lakes, New Jersey, USA) medium for 18 h at 37 °C with shaking (120 rpm) each 2 days. Before the challenge, the bacteria were centrifuged at 2500×*g* for 10 min and washed three times with sterile 0.9% saline solution. The bacteria were resuspended in dinking mineral water (Fonteide®), and bacterial concentration was measured with a spectrometer at 600 nm. Mineral water (Fonteide®) was used to adjust the suspension to 6 × 10^8^ CFU/mL. Finally, the suspension was stored at 4 °C and protected from light.

### *E. coli* Inocula Preparation

The *E. coli* strain used in this study to produce peritonitis was isolated from intestinal microbiota of healthy Sprague Dawley rats from the animal facility of the Hospital Universitario de Gran Canaria, Dr. Negrín. The MALDI-TOF MS (Vitek®MS, Biomerieux) technique [24] was used for the strain identification. The strain was designated as *E. coli* EBETAN-1 HUGCDN. *E. coli* strain cultures were stored at − 80 °C with 20% glycerol (v/v) addition in Brain Heart Infusion broth (PanReac-AppliChem). Fresh cultures were made prior the assays, and the strain was aerobically incubated in Trypticase Soy broth (Becton Dickinson) medium for 18 h at 37 °C with shaking (120 rpm). Before the challenge, the bacteria were centrifuged at 2500×*g* for 10 min and washed three times with sterile 0.9% saline solution. The bacterial concentration was measured with a spectrometer at 600 nm. Sterile 0.9% saline solution was used to adjust the suspension to the desired bacterial concentration (2 × 10^6^ CFU/ml).

### Experimental Design

This study was performed in two phases. First, 30 healthy rats were randomly divided into 6 groups: (i) healthy (negative controls-1), assessed at 7 days (HC7, *n* = 5), 15 days (HC15, *n* = 5), and 30 days (HC30, *n* = 5) after the first dose of tap water, and (ii) animals treated with *A. faecalis* A12C (positive controls-1) and assessed after the first dose of probiotics at 7 days (HA7, *n* = 5), at 15 days (HA15, *n* = 5), and at 30 days (HA30, *n* = 5). Animals in groups HA7, HA15 and HA30 received the probiotic strain *A. faecalis* A12C in the drinking water (Fonteide®) during seven consecutive days. Simultaneously, we administered 2 mL of the same bacterial suspension every 48 h during the first 7 days through an orogastric tube. HC group animals also received the same volume of water (Fonteide®) without probiotic via an orogastric tube. Bottles with the bacterial suspension or only water were replenished daily.

In a second phase, 12 animals were randomly divided into two groups: (i) infected control group (IC) (*n* = 6), in which *A. faecalis* A12C was not administered (negative control-2) and peritoneal infection was induced as described below, and (ii) group infected and pretreated with *A. faecalis* A12C (IA) (*n* = 6), in which the probiotic was administered during the firsts 7 days (positive control-2) in the drinking water (6 × 10^8^ CFU/mL). Simultaneously, 2 mL of the same bacterial suspension or water (Fonteide®) were administered respectively to the IA or the IC groups every 48 h during the first 7 days through an orogastric tube.

**Fig. 1:**
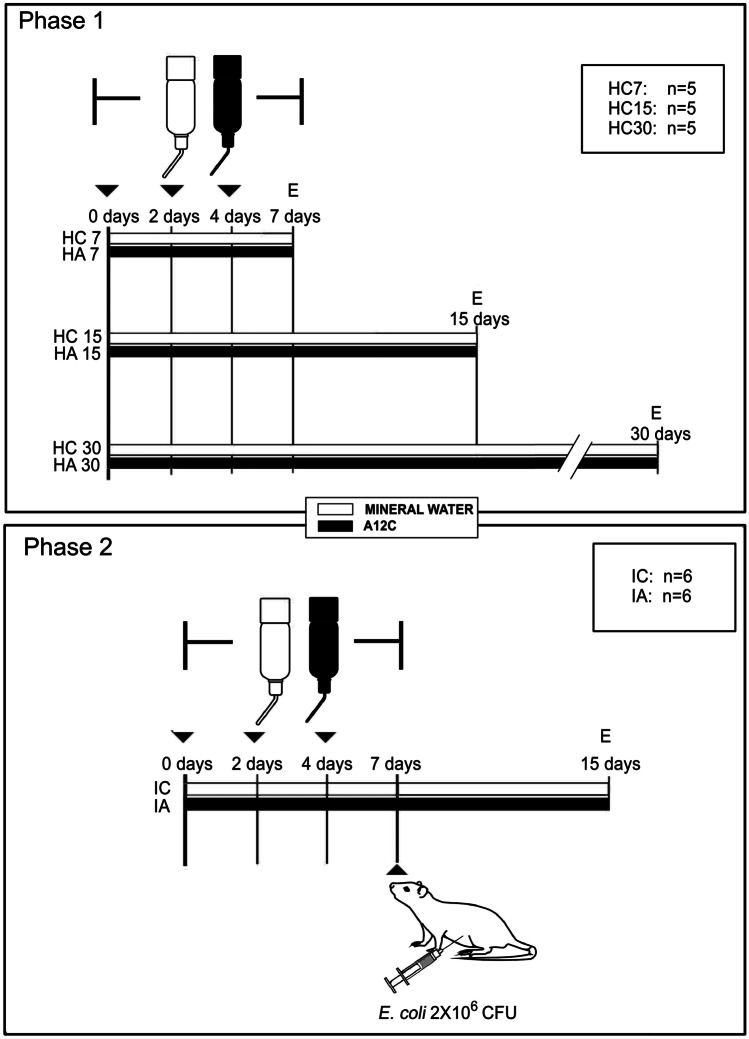
Experimental design and timeline. HC7, HC15, and HC30 Healthy control groups assessed at 7 days, 15 days, and 30 days, respectively. HA7, HA15, and HA30 groups treated with *A. faecalis* A12C and assessed at 7 days, 15 days, and 30 days after the first dose of probiotics. IC Infected Control Group, IA Group treated with *A. faecalis* A12C and infected. Black tip arrow extra orogastric administration of water or *A. faecalis* A12C suspension. E euthanatized

Animals were euthanized at 7, 15, or 30 days after probiotic or mineral water administration (first phase) and at 7 days post *E. coli* inoculation in IA and IC (second phase). Figure 1 summarizes the experimental design.

### Peritonitis Model

For induction of peritonitis, we used a classical model of intraabdominal sepsis [25]. We inoculated an *E. coli* strain isolated from intestinal microbiota of rat as described above. We administered 2 × 10^8^ CFU/animal intraperitoneally using a 25G needle. We delivered analgesia with 0.05 mg/kg buprenorphine/12 h subcutaneous (Buprex®, Mundipharm, Limburg, Germany) during 3 days after the experimental infection. Animals were evaluated every 12 h according to signs shown in Table 1 [26].

**Table 1:** Scoring of rodent protection test

Vital signs	
1. Ruffled fur 2. Weight loss 3. Ocular discharge 4. Lethargy 5. Hunched posture	6. Ataxia 7. Tremor 8. Hypothermia 9. Cyanosis
Conditions	Suggested action
5 + 6 (or 7 or 8 or 9)	Euthanasia

Adapted from Acred et al. [30]

### Body Temperature, Weight, and Sample Collection

At the end of each experimental phase (7th day post-inoculation with *E. coli*, or at 7th, 15th, and 30th days in control animals), animals were weighed; their body temperature was measured in the perianal area using an infrared digital thermometer (T-One. CA-MI srL., Parma, Italy) and anesthetized with 0.3/0.3 mg/kg subcutaneously of a combination of medetomidine (Domtor®, Orion Pharma, Espoo, Finland) and fentanyl (Fentanest®, KERN Pharma, Terrassa, Spain). When animals reached the anesthetic plane, the following samples were collected: blood from external jugular vein for hemogram and biochemistry; intracardiac blood for blood culture and serum collection; urine by direct puncture of urinary bladder for urine culture; bronchoalveolar lavage fluid (BALF) from the right lung (2–3 mL) for microbiological culture; and liver, kidney, spleen, duodenum, pancreas, jejunum, mesenteric lymph nodes, thymus, and lung for histological evaluation. Rats were euthanized by exsanguination after cutting the caudal vena cava and abdominal aorta.

### Blood Count and Blood Chemistry

Hematological parameters (hematocrit, red blood cells, platelets, total white cells, lymphocytes, monocytes, neutrophils, monocytes, eosinophils, and basophils) were counted on whole blood samples in EDTA K3 using an autoanalyser Cell-Dyn Sapphire (Abbott Laboratories, Chicago, Illinois, USA). In venous blood, measurements of serum biochemical parameters, including alanine aminotransferase (ALTL), aspartate aminotransferase (ASTL), creatinine (CREA), urea (UREA), and total bilirubin (TBIL) were performed on a Cobas 8000 Modular Analyser Series (Roche Diagnostics, Basel, Switzerland). Blood obtained by cardiac puncture was centrifuged, and serum was collected and stored at − 80 °C. Serum levels of C-reactive protein (CRP) were measured by ELISA using a rat DuoSet ELISA kit and DuoSet Ancillary Reagent Kit2 (R&D Systems, Abingdon, UK) following manufacturer's protocol.

### Bacterial Counts in Faeces

To determine the survival capability of probiotic *A. faecalis* A12C in the intestine and its influence on the growth of *E. coli* from faecal microbiota, we assessed CFU/g stool of both bacterial species. Faeces were collected from the rectum of animals by abdominal massage at the beginning and at the end of each experimental period. To examine bacterial loads, serial tenfold dilutions were made in 0.9% sterile saline and plated on Brain-Heart infusion Agar (PanReac-AppliChem) modified by adding 4.5 g/100 mL of NaCl, 64 mg/L of vancomycin (SALA, Barcelona, Spain), and 0.02 g/L of bromothymol blue (MERCK, Darmstadt, Germany). After incubation at 37 °C for 24 h (*E. coli*) or 72 h (*A. faecalis* A12C), we calculated total viable counts of original samples (CFU/g faeces). Colonies were separated and isolated 2–3 times. We identified bacterial species using colony morphology and Gram stain. MALDI-TOF MS (Vitek®MS, Biomerieux) technique [24] was used for advanced identification.

### Microbiological Analysis

Samples were processed within 2 h of collection. To investigate aerobic microorganisms, 100 µL of urine, blood, and BALF from each animal were cultured on 10 mL Brain-Heart Infusion broth (PanReac-AppliChem) overnight at 37 °C; 25 µL from these cultures were plated onto CLED, MacConkey, Mannitol Salt agar, and Sabouraud Dextrose Agar (all four from PanReac-AppliChem), and incubated for 24 h at 37 °C. To investigate anaerobic and anaerobic bacteria, 3 mL of blood were inoculated on 10 mL of BD BACTEC Lytic Anaerobic (Becton Dickinson) and incubated in BD BACTEC FX blood culture system (Becton Dickinson) during 5 days. If any sign of growth was detected, a subculture was made on Blood Chocolate and McConkey agar (both of them from Beckton Dickinson), and plates were incubated for 48 h at 37 °C in 5% CO_2_ atmosphere and on Brucella Blood Agar with Hemin and Vitamin K1 (Beckton Dickinson) incubated for 4 days at 37 °C in anaerobiosis. Bacterial species were identified using colony morphology and Gram stain. We used MALDI-TOF MS (Vitek®MS, Biomerieux) for advanced identification.

### Histological Evaluation

Organ samples were fixed in 4% formalin for 24 h, embedded in paraffin, and cut into 4 μ sections for histological study. Slides were stained with hematoxylin-eosin and examined under light microscope. Slides were evaluated by two pathologists blinded to experimental groups. In all organs, the presence of bacteria inside vessels (microscopic observation of blue bacterial plungers inside the vessels, constituted by small rod-shapes), parenchymal disorganization, interstitial edema, leukocyte infiltration, tissue necrosis, and interstitial haemorrhage was evaluated. Lung injury was determined based on alveolar septa thickening and pleuritis. Heart damage was examined if inflammatory response was present in the epicardium and/or myocardium. Intestinal damage was evaluated at mucosal and serosal levels. Peritoneal injury was assessed based on a severity score of histopathological peritonitis [27], as scores of 0, 1, 2, and 3, according to findings in Table 2.

**Table 2:** Histopathology scoring of peritonitis

Score peritonitis	Scoring criteria of histopathologic findings
0	No sign of inflammation or tissue alteration
1	Dilatation of subserosal capillaries, dulling of the peritoneal surface, and swelling of mesothelial cell
2	Thin exudative fibrin film and focal desquamation of mesothelial cells, less than 10 leukocytes per × 60 field
3	Extensive fibrin exudation and diffuse desquamation of mesothelial cells, greater than 10 leukocytes per × 60 field or focal microabscesses

Adapted from Uzunköy et al. [27]

### Statistical Analysis

Analyses were done using Statistical Package R 2019 version 3.5.3 (R Foundation for Statistical Computing, Vienna, Austria). To check the normality of quantitative variables, the Shapiro-Wilk test was used. Continuous variables are expressed as medians and 25th and 75th percentiles. Comparisons between more than two groups were performed using Kruskall-Wallis test, with Bonferroni correction for multiple comparisons when needed. Comparisons between two independent groups were performed using Mann–Whitney *U* test. Data are expressed as box-and whisker diagrams. All tests were two-tailed and statistical significance was considered at *P* < 0.05.

## Results

### *A. faecalis* A12C Safety

Healthy animals pretreated during a week with *A. faecalis* A12C (positive controls-1) and assessed at 7, 15, and 30 days after the first dose of *A. faecalis* A12C (HA7, HA15, and HA30) did not show significant changes in body temperature or weight, compared with negative controls-1 (supplementary data). Survival after *A. faecalis* A12C administration was 100% in all groups. No significant changes were found in most haematological (Table 3) and biochemical (Table 4) parameters. All positive controls-1 (HA7, HA15, HA30) had similar values compared with negative controls-1 (HC) with the exception of blood eosinophils, CREA and ASTL. An increase of eosinophils and CREA at 7 days after *A. faecalis* A12C administration was observed, followed by a progressive normalization. However, a significant decrease of ASTL was observed after the administration of *A. faecalis* A12C which was normalized throughout the study period (Table 3). No histological signs of injury were found in any organ of different groups.

**Table 3:** Hematological parameters at different times post administration of *A. faecalis* A12C

	HC	HA7	HA15	HA30
RBC (× 10^6^/µl)	10.2 (9.8–10.6)	10.2 (10.2–10.3)	10.1 (10–10.1)	9.3 (8.81–10.1)
HGB (g/dl)	17.2 (16.8–17.7)	16.7 (16–17)	16.5 (16.1–17.3)	15.7 (15.3–17.5)
HCT (%)	53.4 (51.97–54.42)	52.1 (48.1–52.1)	52.9 (52.7–52.9)	48.9 (47–54.5)
PLT (× 10^3^/µl)	1011 (894–1030.25)	925 (904–944)	1039 (991–1076)	1011 (1005–1063)
WBC (× 10^3^/µl)	7.91 (7.17–9.17)	8.83 (7.65–9.29)	8.1 (7.57–8.66)	7.21 (6.98 – 7.54)
S (%)	18 (14.7–21.18)	17.1 (16.2–18.5)	15.7 (11.8–16.5)	18.8 (17.6 – 19.1)
Me (%)	0.83 (0.62–1.46)	0.72 (0.56–2.09)	0.86 (0.55–1.02)	0.52 (0.48–0.75)
E (%)	0.32 (0–0.91)	1.5 (1.45–1.82)	0 (0–0.21)	0 (0–0)**
B (%)	1.41 (0.79–2.36)	0.85 (0.5–1.52)	2.3 (1.91–2.87)	0.94 (0.76–1.09)
Le (%)	42 (37.68–44.47)	43.5 (43–47.1)	44.1 (41.4–45.3)	49.5 (45.9–52.4)

The results are expressed as the median (P25–P75) from rats without *A. faecalis* A12C administration (HC, *n* = 15) or 7 days (HA7, *n* = 5), 15 days (HA15, *n* = 5), and 30 days (HA30, *n* = 5) post administration of *A. faecalis* A12C during a week

*RBC* total number of erythrocytes, *HGB* haemoglobin concentration, *HCT* Hematocrit value: erythrocyte ratio of total blood volume, *PLT* total number of platelets, *WBC* total number of leukocytes, *S%* segmented neutrophil percent, *Me%* monocyte percent, *E%* eosinophil percent, *B%* basophil percent, *Le%* lymphocyte percent

^**^P < 0.01 versus HA7

**Table 4:** Serum biochemical parameters at different times post administration of *A. faecalis* A12C

	HC	HA7	HA15	HA30
ASTL (IU/l)	128.5 (120.25–141.25)	86* (82–88)	92^#^ (87–93)	150** (146–156)
ALTL (IU/l)	42 (38.25–44.75)	38 (34–40)	35 (34–36)	33 (33–40)
UREA (mg/dl)	29 (28–31)	29 (27–31)	30 (29–30)	28 (28–29)
CREA (mg/dl)	0.3 (0.29–0.33)	0.35 (0.33–0.35)	0.31 (0.31–0.32)	0.27^#^ (0.27–0.28)
TBIL(mg/dl)	0.7 (0.7–0.8)	0.8 (0.7–0.8)	0.8 (0.8–0.9)	0.5 (0.5–0.7)
CRP (µg/ml)	418.45 (346.27–504.08)	315.81 (278.21–333.21)	410.99 (355.96–24.18)	396.08 (343.83–469.65)

The results are expressed as the median (P25–P75) from rats without *A. faecalis* A12C administration (HC, *n* = 15) or 7 days (HA7, *n* = 5), 15 days (HA15, *n* = 5), and 30 days (HA30, *n* = 5) post administration of *A. faecalis* A12C during a week

*ASTL* aspartate aminotransferase, *ALTL* alanine aminotransferase, *UREA* urea, *CREA* creatinine, *TBIL* total bilirubin, *CRP* C reactive protein

^*^P < 0.05 versus HC, **P < 0.01 versus HA7, ^#^*P* < 0.02 versus HA7

### Gut Colonization Conditions of *A. faecalis* A12C

**Fig. 2:**
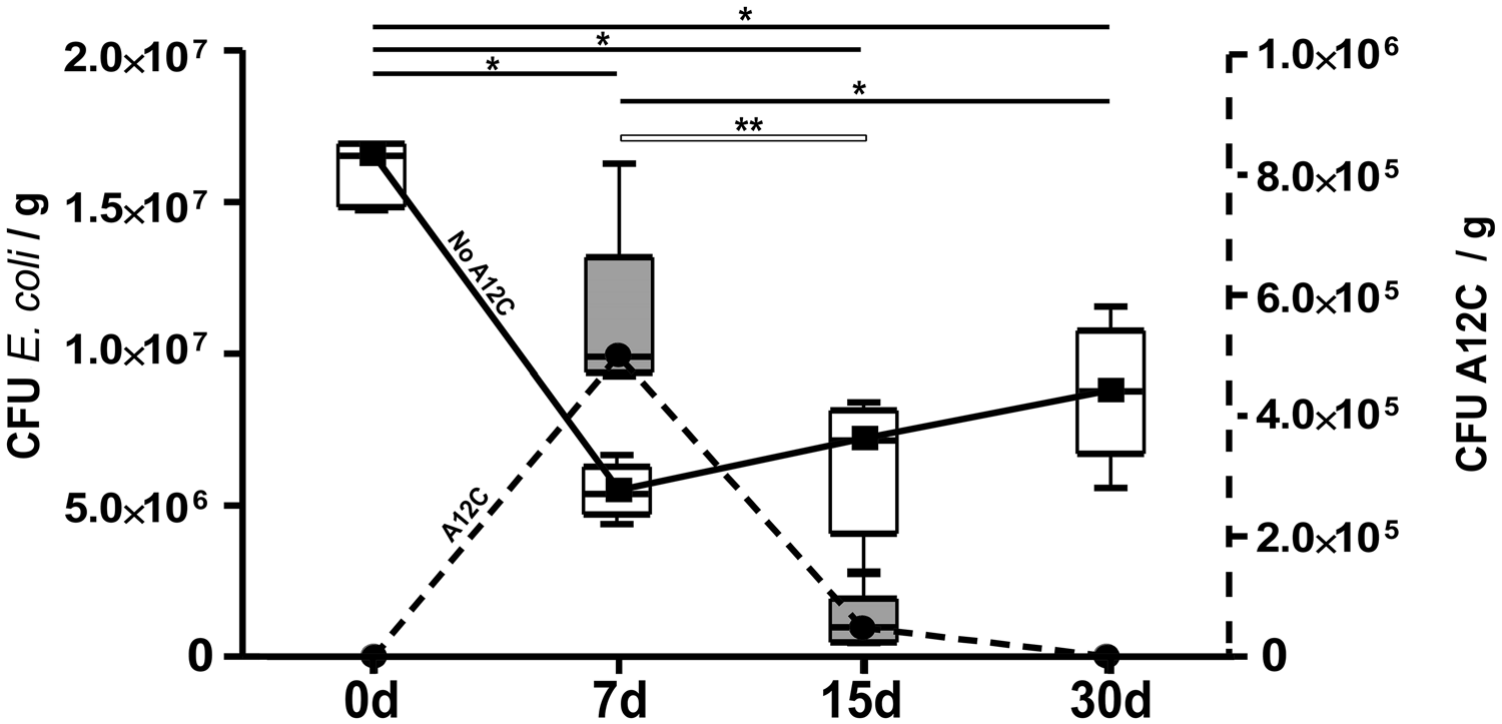
Faeces concentration of *E. coli* and *A. faecalis* A12C at different times in groups treated with *A. faecalis* A12C and assessed at *0d*, 0 days (just before administration of *A. faecalis* A12C), and *7d* 7 days, *15d* 15 days, and *30d* 30 days, after the first dose of probiotic. Box and whisker diagrams depict the smallest observation, lower quartile, median, upper quartile, and largest observation.*P < 0.003, **P < 0.0005

In the positive controls-1 (HA7, HA15, and HA30), concentration of *E. coli* and *A. faecalis* A12C C in faeces showed a significant decrease in *E. coli*, parallel to an increase in *A. faecalis* A12C, 7 days after the administration of this strain (Fig. 2). Subsequently, *E. coli* concentration had a tendency to recover the initial values at 30 days, while *A. faecalis* A12C disappeared from the intestinal microbiota at 30 days.

### Clinical Evolution

**Fig. 3:**
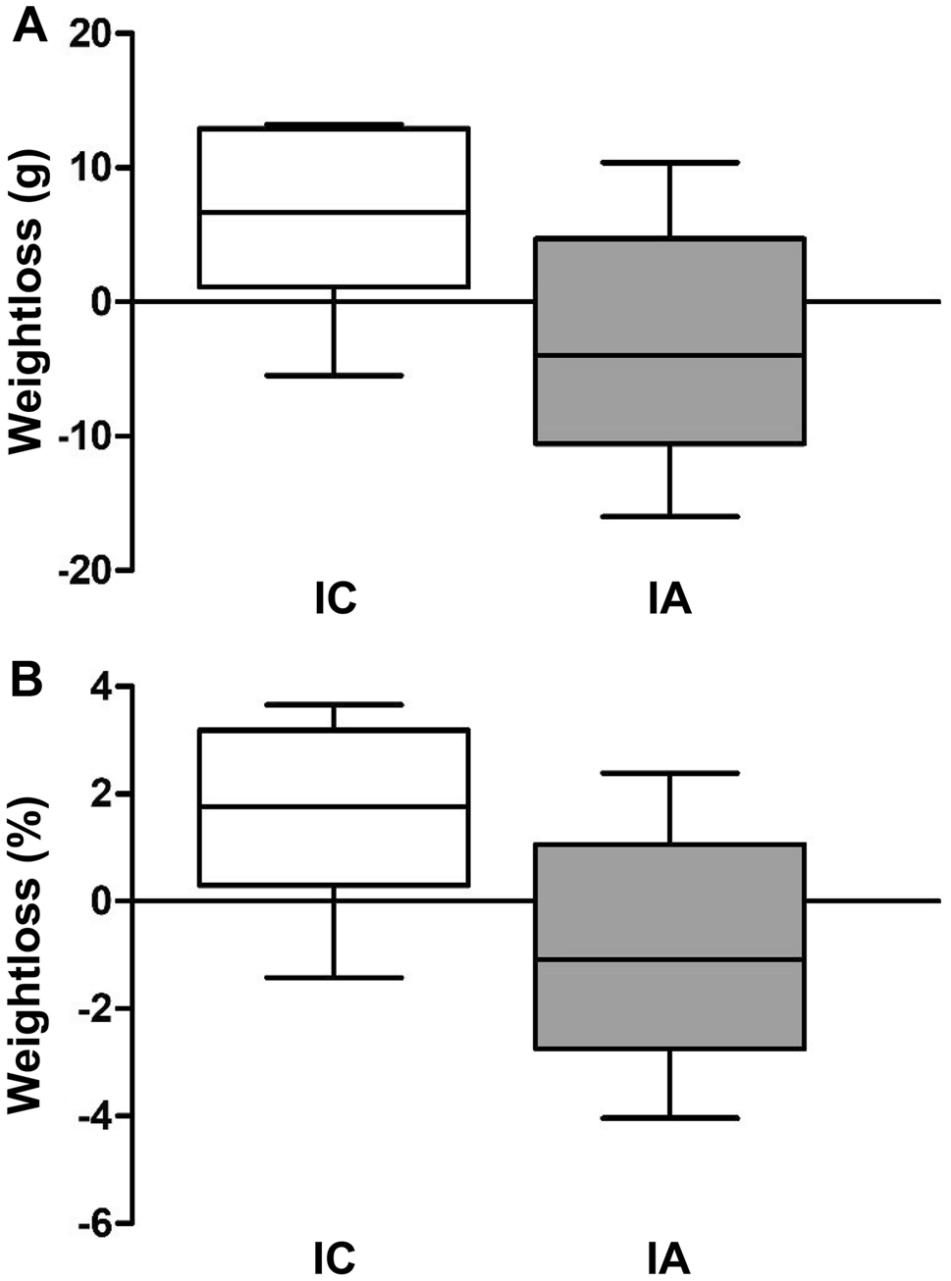
Loss of bodyweight, expressed as difference in grams between weight just in the moment of *E. coli* inoculation (7 days), and at euthanatized time (15 days) **a**, or as percentage of grams lost during the same period of time **b**. *IC* Infected control group, *IA* Infected group pretreated with *A. faecalis* A12C. Box and whisker diagrams depict the smallest observation, lower quartile, median, upper quartile, and largest observation

**Fig. 4:**
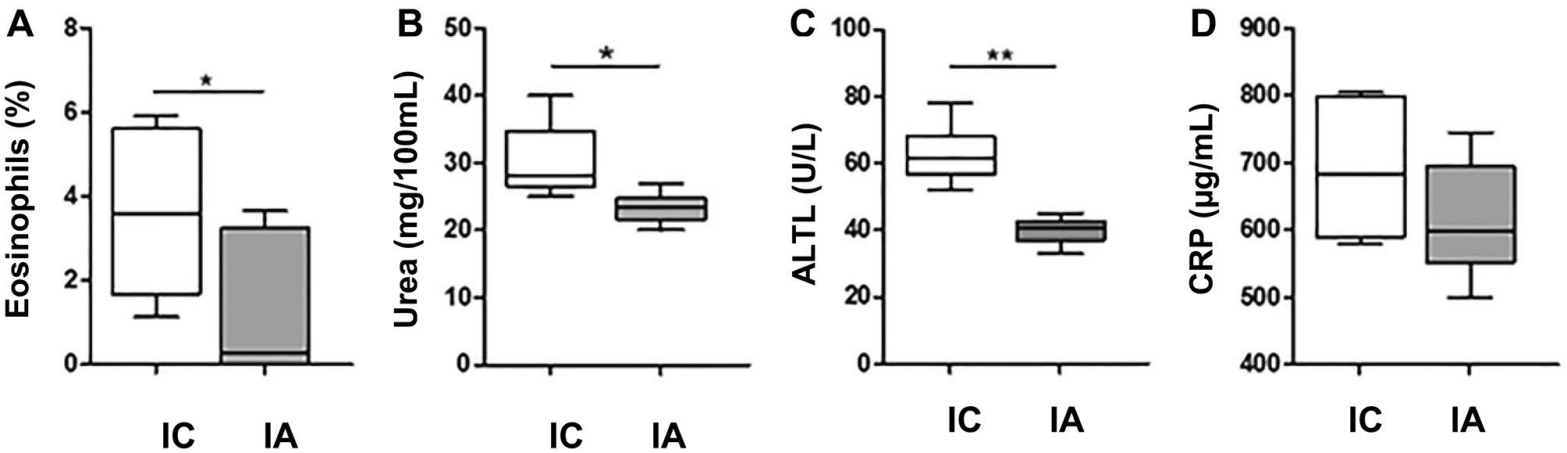
Percentage of eosinophils and serum levels of urea, ALTL (alanine aminotransferase) and CRP (C reactive protein), in Infected Control Group (IC) and Infected group pretreated with *A. faecalis* A12C (IA). **P < 0.01, *P < 0.05

All animals in both groups (IA and IC) had a 100% survival at 15 days after the experiments. However, there was a greater presence of animals with signs of discomfort in IC group (negative control-2) compared with IA group (positive control-2). Ruffled fur was observed in all IC animals, and ocular discharge in 3 of them, while ruffled fur was only observed in 3 animals of the IA group. In both cases, the signs disappeared with analgesic treatment during the first 3 days post-inoculation. In Group IC, a not significant decrease in body weight was observed at day 15 compared with day 7. However, in group IA, a not significant increase was observed in the same period of time (Fig. 3). The only significant changes in serum biochemistry were an increase in ALTL, UREA, and eosinophils in IC respect to IA (Fig. 4).

### Microbiological Control

All animals from IC and IA had negative urine, BAL, and blood cultures at 7 days post-infection with *E. coli*, except for one animal in group IC in which *Aerococcus viridans* and *Staphylococcus xylosus* were isolated in the blood culture.

### Histological Evaluation

**Fig. 5:**
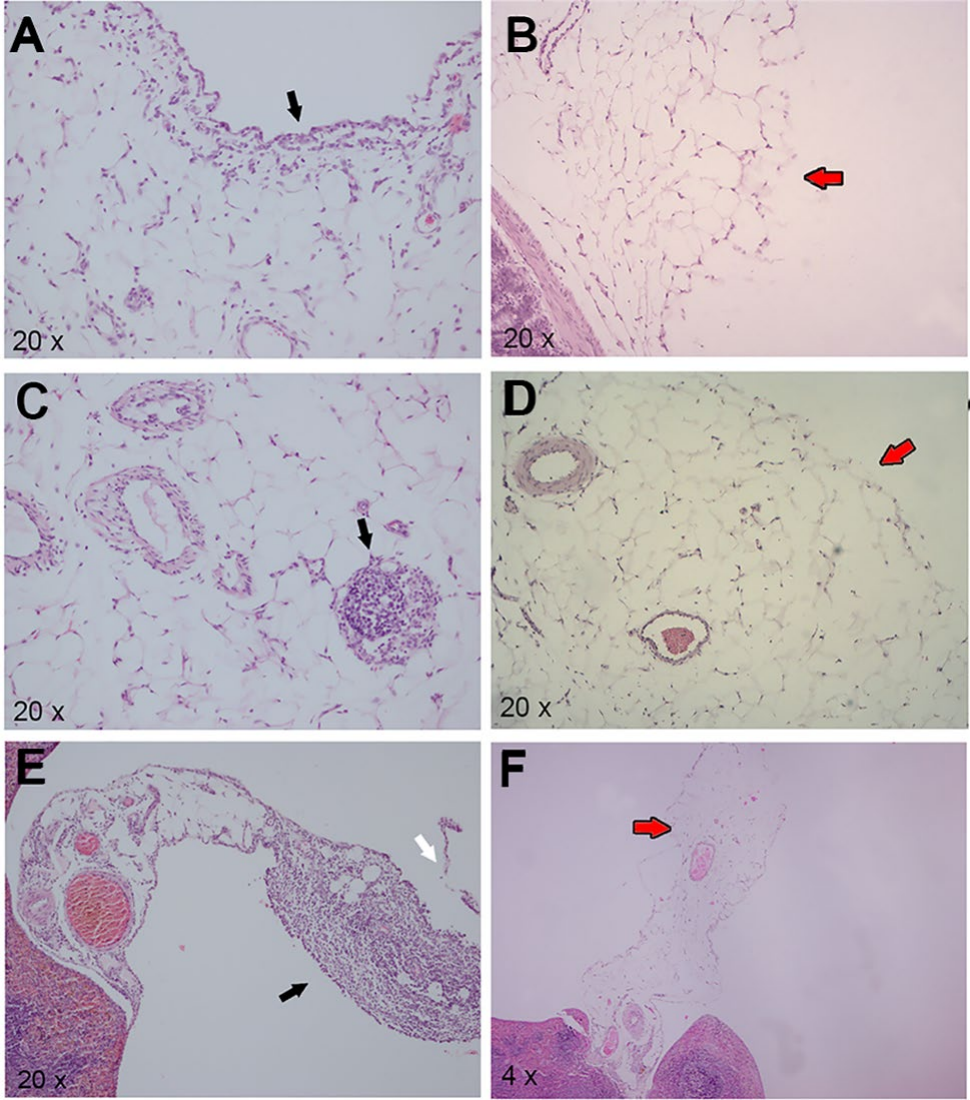
Representative histological images of hematoxilin and eosin stained sections of jejuna peritoneum **a, b**, mesenteric lymph nodes **c, d**, and splenic hilum **e, f**. Dulling of the peritoneal surface, and swelling of mesothelial cell (black arrow) in jejunal peritoneum **a** and splenic hilum peritoneum **e**, plus focal desquamation of mesothelial cells (white arrow) in splenic hilum peritoneum **e**. Mixed polymorphonuclear infiltrate (black arrow) in mesenteric lymphondes **c**, in a rat from IC group. No sign of inflammation or tissue alteration (red arrow) in peritoneal surface of jejunal peritoneum **b**, neither mesenteric lymph nodes **d** nor splenic hilum **f** in a rat from IA group. × 200 magnification **a, b, c, d**, and **e**; × 40 magnification **f**

In the IC group, four animals were classified with a peritonitis score of 1, and two rats with a score of 2. Dilatation of subserosal capillaries, dulling of peritoneal surface, and swelling of mesothelial cells and focal desquamation of mesothelial cells, thin exudative fibrin film, and mild-to-moderate mixed polymorphonuclear infiltrates were observed in these animals. These lesions were more evident in the peritoneum close to mesenteric lymph nodes, splenic hilum, and jejunum. However, lesions compatible with peritonitis were only found in one animal from group IA, which was scored with a value of 1. On other hand, except for the peritoneum, no significant histological changes were observed in any of the organs analysed in both groups. Representative histological lesions are showed in Fig. 5.

## Discussion

The major findings of our study are that *A. faecalis* A12C, isolated from *Argyrosomus regius*, is safe and has marked effects on the spread of infection in a clinically relevant experimental model of peritonitis. To the best of our knowledge, this is the first study exploring the probiotic effects of *A. faecalis* on clinical, biochemical, and pathological parameters in an animal model of peritonitis.

A variety of probiotic species have showed to benefit human and animal health, both in vivo and in vitro [9, 11–13, 16, 28–30]*.* Although lactic acid bacteria have been the most widely used group of probiotics in human and veterinary medicine, studies evaluating other bacterial species with possible probiotic use are increasingly frequent [14–16, 18]. *A. faecalis* is an aerobic, non-fermentative, oxidase-positive, non-encapsulated, Gram-negative rod [31]. It is the most frequently isolated member of the *Alcaligenaceae* family in the clinical laboratory, and it is present in soil and water, in human intestinal microbiota, and in hospital environments [31, 32]. Some strains have been reported as cause of sporadic nosocomial infections [33, 34]. Some studies refer to an eosinophilic response related to alveolitis in humans caused by *A. faecalis* inhalations [35]. Since most probiotic health benefits are strain specific, potential risks are also strain specific. Therefore, generalizing a health risk to probiotics as a class is incorrect, although we do not dispute that there are certain risk groups (e.g., severe immune-compromised patients) where probiotic use should be carefully monitored.

Several experimental and clinical studies reported a decrease in ASTL and ALTL by different probiotics [36–38], but we have not found evidence of an exclusive decrease in ASTL in groups of healthy animals treated with probiotics (positive controls-1) versus healthy animals without administration of probiotics (negative controls-1). It is plausible that *A. faecalis* A12C has a modulating capacity for lipid metabolism, as occurs with other probiotics [39–41]. However, this hypothesis should be evaluated in future studies. CRP is an acute phase protein released in hepatocytes after stimulation of IL-6 and IL-8 in response to acute inflammation, including viral infections, localized bacterial, and other inflammatory processes, and is involved in different immune functions. In both experimental and clinical studies on infectious diseases, it has historically been the reference biomarker of inflammatory response and infection [25, 27, 42]. In our study, CPR values, in conjunction with the absence of positive bacterial blood cultures, support the hypothesis that *A. faecalis* A12C offers safety against a possible spread of this microorganism and a potential risk of sepsis. The observation of a significant decrease in the concentration of *E. coli* after 7 days of administration of *A. faecalis* A12C, to a later recovery to initial concentrations once the administration of the probiotic ceases, is consistent with those reported by other authors [43]. Of note, other studies indicated that *Lactobacillus spp* and inulin supplementation in broilers decreased the numbers of *E. coli* and pH in ileum and cecum [44]. Although the reasons for this suppression are not clear, there are two potential reasons: (i) the decreased bacterial number may result from the inhibition of bacterial proliferation and (ii) a less bacterial infiltration or promoted bacterial killing [9, 45, 46].

Some authors have indicated that the route of infection can affect the host response. An immediate and overwhelming injection of bacteria into the peritoneum or blood compartments initiates rapid and distinct responses. A peritoneal infection will primarily induce inflammatory and immune-cell migration into the infected compartment, whereas a blood-borne model of infection will have the greatest immediate effect on endothelium and vascular system, with subsequent spread to organs. In case of blood inoculation with *E. coli*, rapid changes are noted in serum cytokine levels. However, inoculation of the peritoneal cavity with the same dose of *E. coli* does not generate a robust serum cytokine response [25]. In addition, it has been shown that in infectious peritonitis, the activity of interleukin-10 (IL-10) protects mice from death [47]. Perhaps those effects, together with the slight decrease in the concentration of our *E. coli* inoculums [4], are the reasons behind the zero mortality in our study.

Our model of sepsis was valid to evaluate the progression of sick animals. Infected animals not receiving *A. faecalis* A12C (IC) showed clear signs of discomfort, typical of bacterial infections such as ruffled fur, ocular discharge and weight loss [26]. When comparing haematological and biochemical data of animals pretreated with *A. faecalis* A12C inoculated with *E. coli* (IA), a marked reduction in blood eosinophils, urea, and ALTL occurred. These findings could be related to an anti-inflammatory and/or immunomodulatory capacity of *A. faecalis* A12C. Although we did not find inflammatory histopathological lesions in kidney or liver, we found moderate inflammation with polymorphonuclear infiltrates in the peritoneum and fatty tissue in rats from infected control group (negative control-2) or IC, which were significantly reduced in IA animals (positive control-2). This inflammatory improvement at the histological level has also been demonstrated with similar models of peritonitis evaluating different therapies, where the thickness of the peritoneal surface, the desquamation of the mesothelial cells, and the infiltrates of inflammatory cells were evaluated [27]. ALTL is more specific for liver damage than ASTL, because the former is located almost exclusively in the cytosol of the hepatocyte, while ASTL, in addition to the cytosol and mitochondria, is found in the heart, skeletal muscle, kidneys, brain, pancreas, lung, erythrocytes, and leukocytes [48]. The elevations in ALTL and urea in our study correlate with those obtained by other authors in similar models of peritonitis with or without probiotic evaluation. [13, 49]. These biochemical and haematological changes corresponded to an increase in CRP in IC when compared with IA groups. Our data suggest a potential anti-inflammatory/immunomodulatory effect of *A. faecalis* A12C.

The anti-inflammatory potency at the intestinal level of probiotics has been explained in experimental studies by several mechanisms [50], including suppression of apoptosis, epithelial cell proliferation and migration, increase colonic IL-10, or repair of disruptions of enteric neurotransmissions. β-glucans are glucose polymers of high molecular weight naturally present in the cell wall of living organisms such as bacteria, yeast, fungus, and plants. They are recognized by the innate immune system. This recognition plays important roles in host defense and represents specific opportunities for clinical modulation of the host immune response [51]. Some authors have examined the effects of curdlan, a β-(1-3)- glucan derived from *A. faecalis*, on allergic airway inflammation using a murine model of asthma, and suggest that curdlan is capable of inducing IL-10-producing CD4+ T cells and inhibiting the development of eosinophilic airway inflammation [52]. This finding could have some similarity with the decrease of eosinophils in IA animals.

Our study has some limitations and strengths. First, a higher sample size could provide more robust results. Second, we do not know the possible effects that *A. faecalis* A12C may cause in animals after 7 days of its administration and the safety and efficacy at different doses. Third, although the use of a semiquantitative scoring system, as the one used in this study for histological evaluation, is quite common, we are aware that such score does not provide a fully quantitative assessment of histopathological alterations, as a morphometrical assessment would do. However, the major strength of this study is that we used a translational model of peritonitis that allowed us to obtain results of clinical relevance with a global vision of the potential therapeutic role of an *A. faecalis* strain.

In summary, our findings suggest that *A. faecalis* A12C could influence clinically relevant parameters in sepsis. Administration of 6 × 10^8^ CFU/mL for 7 days was associated with a lesser spread of infection. Our study may add a piece to the complex puzzle of bacteria-host interactions and new therapeutic possibilities in the prophylaxis of sepsis. However, future studies are necessary to analyse these parameters in more detail.

## Supplementary Information

Below is the link to the electronic supplementary material.

Supplementary file1 (DOCX 21 KB)

## Data Availability

The datasets generated and/or analysed during the current study are available from the corresponding author on reasonable request.
