# Supplementary material for: Probiotic Properties of Alcaligenes faecalis Isolated from Argyrosomus regius in Experimental Peritonitis (Rat Model)
Source: Probiotics Antimicrob Proteins. 2021 Mar 13;13(5):1326–37. doi: 10.1007/s12602-021-09767-7 (PMC8463381; doi:10.1007/s12602-021-09767-7)
Supplement: Supplementary file 1 — Supplementary file1 (DOCX 21 KB) [file 12602_2021_9767_MOESM1_ESM.docx]

SUPPLEMENTARY RESULTS

**Probiotic properties of *Alcaligenes faecalis* isolated from *Argyrosomus regius* in experimental peritonitis**

**^1^AI Gutiérrez-Falcón** (ORCID iD: 0000-0002-2672-9356)

**^2,3^AM Ramos-Nuez**

**^4^A Espinosa de los Monteros y Zayas** (ORCID iD: 0000-0002-7736-3139)

**^1^DF Padilla Castillo** (ORCID iD: 0000-0002-6678-5029)

**^2,3^M Isabel García-Laorden** (ORCID iD: 0000-0001-6270-6306)

**^5^FJ Chamizo López** (ORCID iD: 0000-0003-1328-1924)

**^1^F Real Valcárcel** (ORCID iD: 0000-0001-6526-0354)

**^5^F Artilles Campelo** (ORCID iD: 0000-0003-3019-8604)

**^5^A Bordes Benítez** (ORCID iD: 0000-0003-3243-7402)

**^6^P Nogueira Salgueiro** (ORCID iD: 0000-0002-8029-2685)

**^6^C Domínguez Cabrera**

**^7^JC Rivero-Vera**

**^8^JM González-Martín** (ORCID iD: 0000-0001-6816-4157)

**^9^J Martín Caballero** (ORCID iD: 0000-0002-1579-2739)

**^10^R Frías Beneyto** (ORCID iD: 0000-0001-7569-5693)

**^2, 3^Jesús Villar** (ORCID iD: 0000-0001-5687-3562)

**^* 1,3,11^JL Martín-Barrasa,** (ORCID iD: 0000-0002-3280-9838)

*^1^Animal Infectious Diseases and Ictiopathology, University Institute of Animal Health and Food Safety, University of Las Palmas de Gran Canaria, Carretera de Trasmontaña s/n, 35416 Arucas, Spain.*

*^2^CIBER de Enfermedades Respiratorias, Instituto de Salud Carlos III, Monforte de Lemos 3-5, Pabellón 11, 28029 Madrid, Spain.*

*^3^Multidisciplinary Organ Dysfunction Evaluation Research Network, Research Unit, Hospital Universitario de Gran Canaria Dr. Negrín, Barranco de la Ballena s/n, 35019 Las Palmas de Gran Canaria, Spain.*

*^4^Morphology Department. Universitary Institute of Animal Health and Food Safety (IUSA).Universidad de Las Palmas de Gran Canaria. Arucas. Las Palmas. Spain.*

*^5^Microbiology Department. Hospital Universitario de Gran Canaria Dr Negrín, Barranco de la Ballena s/n, 35019 Las Palmas de Gran Canaria, Spain.*

*^6^Clinical Biochemistry Department. Hospital Universitario de Gran Canaria Dr Negrín, Barranco de la Ballena s/n, 35019 Las Palmas de Gran Canaria, Spain.*

*^7^Pathology Service. Hospital Universitario de Gran Canaria Dr Negrín, Barranco de la Ballena s/n, 35019 Las Palmas de Gran Canaria, Spain.*

*^8^Statistics Service. Research Unit, Hospital Universitario de Gran Canaria Dr Negrín, Barranco de la Ballena s/n, 35019 Las Palmas de Gran Canaria, Spain.*

*^9^Barcelona Biomedical Research Park (PRBB), Barcelona, Spain.*

*^10^Comparative Medicine, Karolinska Institutet, Stockholm, Sweden*

*^11^Animal Facility, Research Unit, Hospital Universitario de Gran Canaria Dr Negrín, Barranco de la Ballena s/n, 35019 Las Palmas de Gran Canaria, Spain.*

**∗Corresponding author:** JL Martín-Barrasa, Research Unit, Hospital Universitario de Gran Canaria, Dr. Negrín, Barranco de la Ballena s/n, 35019 Las Palmas de Gran Canaria, Spain. Phone: +(34) 928 449277 / +(34) 650 056569. E-mail address: joseluis.martin@ulpgc.es

**Supplementary table 1.** Body temperature at different times in groups treated (positive controls-1) or no-treated (negative controls-1) with *A. faecalis* A12C.

| **Groups** | **Temperature (ºC)** |
| --- | --- |
| **HC0** | 35.4 (35.2-35.8) |
| **HA0** | 35.2 (34.7-36) |
| **HC7** | 35.3 (34.6-36) |
| **HA7** | 34.9 (34.8-35) |
| **HC15** | 35.4 (34.3-35.6) |
| **HA15** | 35.1(35.1-35.4) |
| **HC30** | 34.7 (34.6-35.3) |
| **HA30** | 35.4 (35.1-36) |

The results are expressed as the median (P25-P75) of the body temperature. HC0, HC7, HC15 and HC30: Healthy control groups (negative controls-1) assessed at 0 days, 7days, 15 days and 30 days respectively. HA0, HA7, HA15 and HA30 groups treated with *A. faecalis* A12C (positive controls-1) and assessed at 0 days, 7days, 15 days and 30 days after the first dose of probiotics respectively.

**Supplementary table 2.** Bodyweight at different times in groups treated (positive controls-1) or no-treated (negative controls-1) with *A. faecalis* A12C .

| **Groups** | **Bodyweight (g)** |
| --- | --- |
| **HC7** | 418.6 (394.5-428.5) |
| **HA7** | 418.4 (388.1-421.1) |
| **HC15** | 427.2 (401.5-433.8) |
| **HA15** | 428.8 (390.5-437.3) |
| **HC30** | 453.8 (422.9-460.5) |
| **HA30** | 455,9 (449,5-465,2) |

The results are expressed as the median (P25-P75) of the bodyweight. HC7, HC15 and HC30: Healthy control groups (negative controls-1) assessed at 7days, 15 days and 30 days respectively. HA7, HA15 and HA30 groups treated with *A. faecalis* A12C (positive controls-1) and assessed at 7days, 15 days and 30 days after the first dose of probiotics respectively.

**Supplementary table 3.** Bodyweight at the moment of *E. coli* inoculation and at euthanasia time

in infected animals no pretreated, or pretreated, with *A. faecalis* A12C .

|  | **IC** | | **IA** | |
| --- | --- | --- | --- | --- |
|  | **7D** | **15D** | **7D** | **15D** |
| **Weight (g)** | 382.40  (363.73-391.18) | 379.95  (358.40-390.63) | 371.00  (366.00-396.00) | 372.00  (370.00-412.00) |
| **D7-D15 (g)** | 6.65 (3.98-11.43) | | -4.00 (-5.10 - -1.00) | |
| **D7-D15 (%)** | 1.77 (1.07-2.74) | | -1.09 (-1.45 - -0.27) | |

The results are expressed as the median (P25-P75) of the bodyweight in grams, or the median (P25-P75) of the difference in the bodyweight (D7-D15) between the moment of *E. coli* inoculation (7 days) and at euthanasia time (15 days) in grams and percentage of grams.

IC Infected control group (negative control-2), IA Infected group pretreated with *A. faecalis* A12C (positive control-2)
